# Physicians’ Attitudes Toward Telemedicine Consultations During the COVID-19 Pandemic: Cross-sectional Study

**DOI:** 10.2196/29251

**Published:** 2021-06-01

**Authors:** Noora Alhajri, Mecit Can Emre Simsekler, Buthaina Alfalasi, Mohamed Alhashmi, Majd AlGhatrif, Nahed Balalaa, Maryam Al Ali, Raghda Almaashari, Shammah Al Memari, Farida Al Hosani, Yousif Al Zaabi, Shereena Almazroui, Hamed Alhashemi, Ovidiu C Baltatu

**Affiliations:** 1 Khalifa University College of Medicine and Health Science Abu Dhabi United Arab Emirates; 2 College of Engineering Khalifa University Abu Dhabi United Arab Emirates; 3 Department of Family Medicine Zayed Military Hospital Abu Dhabi United Arab Emirates; 4 Department of Medicine The Johns Hopkins University School of Medicine Baltimore, MD United States; 5 Department of General Surgery Sheikh Shakhbout Medical City Abu Dhabi United Arab Emirates; 6 Ambulatory Health Services, Zafarana Clinic Abu Dhabi Healthcare Company Abu Dhabi United Arab Emirates; 7 Department of Dermatology Sheikh Khalifa Medical City Abu Dhabi United Arab Emirates; 8 Abu Dhabi Public Health Center Department of Health Abu Dhabi United Arab Emirates; 9 Department of Health Abu Dhabi United Arab Emirates

**Keywords:** audio consultation, clinical decision-making, clinical training, communication, COVID-19, outpatient department, perception, telemedicine, United Arab Emirates, video consultation

## Abstract

**Background:**

To mitigate the effect of the COVID-19 pandemic, health care systems worldwide have implemented telemedicine technologies to respond to the growing need for health care services during these unprecedented times. In the United Arab Emirates, video and audio consultations have been implemented to deliver health services during the pandemic.

**Objective:**

This study aimed to evaluate whether differences exist in physicians’ attitudes and perceptions of video and audio consultations when delivering telemedicine services during the COVID-19 pandemic.

**Methods:**

This survey was conducted on a cohort of 880 physicians from outpatient facilities in Abu Dhabi, which delivered telemedicine services during the COVID-19 pandemic between November and December 2020. In total, 623 physicians responded (response rate=70.8%). The survey included a 5-point Likert scale to measure physician’s attitudes and perceptions of video and audio consultations with reference to the quality of the clinical consultation and the professional productivity. Descriptive statistics were used to describe physicians’ sociodemographic characteristics (age, sex, designation, clinical specialty, duration of practice, and previous experience with telemedicine) and telemedicine modality (video vs audio consultations). Regression models were used to assess the association between telemedicine modality and physicians’ characteristics with the perceived outcomes of the web-based consultation.

**Results:**

Compared to audio consultations, video consultations were significantly associated with physicians’ confidence toward managing acute consultations (odds ratio [OR] 1.62, 95% CI 1.2-2.21; *P*=.002) and an increased ability to provide patient education during the web-based consultation (OR 2.21, 95% CI 1.04-4.33; *P*=.04). There was no significant difference in physicians’ confidence toward managing long-term and follow-up consultations through video or audio consultations (OR 1.35, 95% CI 0.88-2.08; *P*=.17). Video consultations were less likely to be associated with a reduced overall consultation time (OR 0.69, 95% CI 0.51-0.93; *P*=.02) and reduced time for patient note-taking compared to face-to-face visits (OR 0.48, 95% CI 0.36-0.65; *P*<.001). Previous experience with telemedicine was significantly associated with a lower perceived risk of misdiagnosis (OR 0.46, 95% CI 0.3-0.71; *P*<.001) and an enhanced physician-patient rapport (OR 2.49, 95% CI 1.26-4.9; *P*=.008).

**Conclusions:**

These results indicate that video consultations should be adopted frequently in the new remote clinical consultations. Previous experience with telemedicine was associated with a 2-fold confidence in treating acute conditions, less than a half of the perceived risk of misdiagnosis, and an increased ability to provide patients with health education and enhance the physician-patient rapport. Additionally, these results show that audio consultations are equivalent to video consultations in providing remote follow-up care to patients with chronic conditions. These findings may be beneficial to policymakers of e-health programs in low- and middle-income countries, where audio consultations may significantly increase access to geographically remote health services.

## Introduction

The COVID-19 pandemic has caused an enormous burden on the health care system and health care delivery worldwide [1-4]. As social distancing and quarantining became the new normal, face-to-face clinical visits plummeted, causing the health care system to rapidly shift to telemedicine to leverage their response to the pandemic [5-8]. Telemedicine created new opportunities for patient care in the context of the COVID-19 pandemic and thus reduced health care disparities [9,10]. Telemedicine is available in various modalities including patient portals, emails, text messages, telemonitoring, store-and-forward, audio consultations, and real-time video consultations [10-13]. The wide variety in communication channels offer different opportunities for providers to manage patients who are in quarantine or live in remote areas, which reduces the risk of disease transmission and improves access to health care services [5,9,14,15].

Owing to the growing concern regarding the risk of workplace transmission, the use of telemedicine services increased globally [16-19], and the United Arab Emirates is no different. In March 2020, Abu Dhabi launched its first Telemedicine Virtual Outpatient Clinic to support the continuity of patient care [20]. It has been estimated that within only 1 month, physicians across Abu Dhabi SEHA hospitals performed over 28,000 virtual consultations [21,22].

Studies conducted on telemedicine during the COVID-19 pandemic, while yielding meaningful insights on its role, have largely been based on physician knowledge of telemedicine in specific subspecialities and have been limited to descriptive data of certain encounters rather than quantifying their association. Currently, the effect of video vs audio consultations on physicians’ attitude toward telemedicine is unclear [23,24]. Moreover, barriers against its full implementation beyond the context of the COVID-19 pandemic remain unexplored. Identifying these barriers within each modality, which prevent their successful adoption by health care providers, is essential for directing future infrastructure to modernize the health care system and improve telemedicine utilization and outcomes. This study aimed to describe physicians’ attitudes toward the use of telemedicine services in Abu Dhabi during the COVID-19 pandemic. We also aimed to explore the effects of audio vs video consultations and physicians’ sociodemographic characteristics on their confidence during the clinical consultation, perceived quality of care, and perceived effects of professional productivity. Future studies are needed to objectively assess the effect of telemedicine modalities on the quality of care and professional productivity and to guide future infrastructure investments to assure embracing this new opportunity to provide high-quality health care to a larger number of patients in the post–COVID-19 era.

## Methods

### Study Design and Ethics Approval

This was a survey-based study conducted on physicians in outpatient facilities in Abu Dhabi, which provided telemedicine services during the COVID-19 pandemic between November and December 2020. Ethics approval was obtained from the institutional review board of Khalifa University (protocol# H21-006-2020) and of the Abu Dhabi COVID-19 Research Committee of the Department of Health in Abu Dhabi (reference# DOH/CVDC/2020/1747). Surveys were administered through the Department of Health and SEHA, these being the major health authorities in Abu Dhabi. The institutional review board or ethics committee at each participating institution approved the study protocol and survey. Electronic written consent was waived for this data-only study owing to the deidentified nature of this survey. The present study followed the STROBE (Strengthening the Reporting of Observational Studies in Epidemiology) reporting guidelines for cross-sectional studies [25].

### Subject Selection and the Inclusion and Exclusion Criteria

The survey was administered to a cohort of 880 physicians at outpatient facilities in Abu Dhabi, who met the following inclusion criteria: being a physician practicing at an outpatient facility in Abu Dhabi and providing audio or video consultations during the COVID-19 pandemic from January to November 2020. Exclusion criteria were being of another allied health care profession such as nurses, pharmacists, and technicians (as our study targeted physicians only) or physicians who did not work at outpatient departments and who did not use telemedicine during the COVID-19 pandemic. From a total of 880 physicians listed, 623 responded to the survey (response rate=70.8%).

### Survey Development, Piloting, and Data Collection

A web-based structured survey containing multiple-choice questions was developed by reviewing published telemedicine surveys and their instruments [26-28]. The web-based survey had 6 components, which contained a total of 42 questions related to physicians’ perceptions and attitudes toward telemedicine. A pilot survey was conducted, which included a cohort of 25 physicians in Abu Dhabi, who frequently used telemedicine during the COVID-19 pandemic. The main web-based survey was developed using the Microsoft Forms platform (Microsoft Corp) and was sent to the physicians at outpatient facilities via the hospital’s internal email system. To reduce the risk of attrition bias, we ensured generating a good rapport between on-site principal investigators and the study participants by sending customized invitations [29,30]. Furthermore, a follow-up email was sent 1 week apart from the initial date of survey distribution to remind nonresponders to participate in the survey.

### Study Variables and Outcomes

This was a self-administered survey that gathered data on physicians’ sociodemographic characteristics including age, sex, telemedicine modality, clinical specialty, designation, number of years in practice, and past experience with telemedicine. We also gathered data using a 5-point Likert scale to assess (1) physicians’ current experience with telemedicine, (2) perceived quality of the web-based clinical consultation, (3) satisfaction with telemedicine, (4) perceived professional productivity compared to traditional face-to-face visits, (5) willingness to use telemedicine after the pandemic, and (6) perceived barriers to telemedicine use. Data on these 6 components were gathered to understand the telemedicine experience better during the COVID-19 pandemic and to gain insights into the preparedness of the digital health care response for any potential crisis. We defined “acute remote care consultation” as any remote consultation made for the first time owing to an urgent medical complaint, the onset of a new disease, or a follow-up case that has not received a consultation for more than 6 months. Furthermore, “chronic remote care consultation” was defined as any remote follow-up consultation within 6 months of the initial in-person visit for a long-term medical condition [31].

### Statistical Analysis

Differences between video and audio consultations were investigated using various outcome variables, which included 2 main parts. While the first set of outcomes was related to the perceived quality of clinical consultations, the second set of outcomes tested physicians’ professional productivity with telemedicine over face-to-face consultations.

Descriptive statistics characterizing the study cohort were reported as frequency and percentage values for all variables. To compare the responses to our survey questions with regard to video and audio consultations, we performed chi-square analysis at a significance level of .05.

We used ordered logistic regression analyses to investigate the association between outcome variables and the modality, adjusting for confounding factors such as sociodemographic characteristics. A forced-entry approach was adopted to consider the variance inflation factor (VIF) diagnostic to prevent obtaining unreliable estimates of coefficients and odds ratios (ORs) owing to high correlations among predictor variables. Considering the high VIF for the variable of the number of years in practice (VIF>4), we excluded this variable and confirmed that multicollinearity is not a concern in the final models (VIF=1.51). Further, the Akaike information criterion was used to assess the fit of the models after excluding the variable of the number of years in practice. Survey questions were answered on a 5-point Likert scale where 5=“strongly agree”, 4=“agree”, 3=“neutral”, 2=“disagree”, and 1=“strongly disagree”. However, owing to limited observations toward the extreme ends of the scale (“strongly agree” and “strongly disagree”), we merged the responses of “strongly agree” and “agree” under positive responses and “strongly disagree” and “disagree” under negative responses together as these 2 statements were found to involve the same attitude continuum toward the question [32]; these were grouped under “disagreement,” “neutral,” and “agreement.” The outcomes of the regression models were reported as ORs with 95% CI values, and *P*<.05 indicated significance. Statistical analyses were performed using STATA (version 16.1, Stata Corp).

## Results

Overall, 623 physicians completed the survey, of whom 347 (55.7%) conducted only audio consultations and 276 (44.3%) conducted only video consultations during the COVID-19 pandemic. The sociodemographic descriptive characteristics of the 2 groups are summarized and compared in Table 1.

**Table 1:** Sociodemographic characteristics of the physicians included in the study (N=623) and descriptive statistics by modality.

Sociodemographic characteristics		Audio consultations (n=347), n (%)	Video consultations (n=276), n (%)	Total, n (%)	*P* value	
**Sex**						.04
	Female	163 (46.97)	107 (38.77)	270 (43.34)		
	Male	184 (53.03)	169 (61.23)	353 (56.66)		
**Age (years)**						.52
	≤39	87 (25.07)	59 (21.38)	146 (23.43)		
	40-49	138 (39.77)	116 (42.03)	254 (40.77)		
	50-59	83 (23.92)	62 (22.46)	145 (23.27)		
	≥60	39 (11.24)	39 (14.13)	78 (12.52)		
**Specialty**						.23
	Internal medicine	213 (61.38)	186 (67.39)	399 (64.04)		
	Surgical specialties	38 (10.95)	22 (7.97)	60 (9.63)		
	Family medicine	76 (21.90)	48 (17.39)	124 (19.90)		
	Others^a^	20 (5.76)	20 (7.25)	40 (6.42)		
**Physician designation**						.13
	General physician	62 (17.87)	41 (14.86)	103 (16.53)		
	Resident	8 (2.31)	3 (1.09)	11 (1.77)		
	Specialist	189 (54.47)	175 (63.41)	364 (58.43)		
	Consultant	88 (25.36)	57 (20.65)	145 (23.27)		
**Number of years in practice**						.32
	≤4	16 (4.61)	10 (3.62)	26 (4.17)		
	5-9	52 (14.99)	33 (11.96)	85 (13.64)		
	10-20	132 (38.04)	124 (44.93)	256 (41.09)		
	>20	147 (42.36)	109 (39.49)	256 (41.09)		
**Past experience with telemedicine**						.09
	Never used	256 (73.78)	219 (79.35)	475 (76.24)		
	Used a few times	75 (21.61)	41 (14.86)	116 (18.62)		
	Used frequently	16 (4.61)	16 (5.80)	32 (5.14)		

^a^Other specialties include speech therapy, dentistry, physical medicine and rehabilitation, anesthesiology, emergency medicine, occupational therapy, radiology, aviation and occupational health, periodontics, gynecology center, nutrition, urgent care, prosthodontics, and critical care medicine.

### Sociodemographic Characteristics

Compared to physicians who provided audio consultations, those who provided video consultations were predominantly male (61.23% vs 53.03%, respectively; *P*=.04), middle-aged (40-49 years: 42.03% vs 39.77%, 50-59 years: 22.46% vs 23.92%, ≥60 years: 14.13% vs 11.24%; *P*=.52), and had a different specialty distribution with most belonging to internal medicine subspecialities (67.39% vs 61.38%; *P*=.23). Additionally, physicians who provided video consultations were mostly specialists with 10-20 years of experience in practice. In relation to previous experience with telemedicine modalities, there was a variation in responses. The majority of physicians who provided video consultations during the COVID-19 pandemic reported that they had never used this form of telemedicine previously, compared to their counterparts who provided audio consultations (79.35% vs 73.78%, respectively; *P*=.09); conversely, the proportion of physicians who reported frequent provision of video consultations was higher than that of their counterparts who provided audio consultations (5.80% vs 4.61%; *P*=.09).

### Perceived Quality of Clinical Care Provided

Physicians’ agreement with the following statements was assessed: (1) I was confident in managing acute conditions, (2) I was confident in managing chronic conditions, (3) I was able to answer my patients’ questions, (4) I was able to provide health education to patients, and (5) I had an impression of misdiagnosis risk during the teleconsultation. The proportions of physicians who agreed, disagreed, or were neutral about the statements are indicated in Table 2**.** Overall, more than half of the physicians who provided video consultations agreed that they were confident in diagnosing acute conditions (*P*=.01), confident in diagnosing chronic conditions (*P*=.08), and able to provide patient health education during the clinical consultation, which was significantly higher than that of physicians who provided audio consultations (*P*=.006). However, there was no significant difference in the perceived risk of misdiagnosis (*P*=.41) and the physicians’ ability to address the patients’ questions (*P*=.26) among those who provided video or audio consultations. Remarkably, the proportion of male physicians who believed that telemedicine raises the likelihood of misdiagnosis was higher than the proportion of female physicians (*P*=.02) (Multimedia Appendix 1).

**Table 2:** Comparison of survey responses on the perceived quality of clinical care provided by modality.

Perceived quality of clinical care provided			Audio consultations, n (%)		Video consultations, n (%)		Total, n (%)		*P* value	
**Confidence in managing acute consultations**										.01
	Disagree and strongly disagree	85 (24.50)		47 (17.03)		132 (21.19)				
	Neutral	121 (34.87)		85 (30.80)		206 (33.07)				
	Agree and strongly agree	141 (40.63)		144 (52.17)		285 (45.75)				
**Confidence in managing chronic conditions and follow-up consultations**										.08
	Disagree and strongly disagree	20 (5.76)		6 (2.17)		26 (4.17)				
	Neutral	50 (14.41)		39 (14.13)		89 (14.29)				
	Agree and strongly agree	277 (79.83)		231 (83.70)		508 (81.54)				
**Ability to answer patients’ questions**										.26
	Disagree and strongly disagree	9 (2.59)		3 (1.09)		12 (1.93)				
	Neutral	36 (10.37)		23 (8.33)		59 (9.47)				
	Agree and strongly agree	302 (87.03)		250 (90.58)		552 (88.60)				
**Ability to provide patient health education**										.006
	Disagree and strongly disagree	15 (4.32)		1 (0.36)		16 (2.57)				
	Neutral	36 (10.37)		24 (8.70)		60 (9.63)				
	Agree and strongly agree	296 (85.30)		251 (90.94)		547 (87.80)				
**Perceived risk of misdiagnosis with telemedicine**										.41
	Disagree and strongly disagree	47 (13.54)		35 (12.68)		82 (13.16)				
	Neutral	84 (24.21)		80 (28.99)		164 (26.32)				
	Agree and strongly agree	216 (62.25)		161 (58.33)		377 (60.51)				

### Perceived Professional Productivity

The overall response to this survey section varied across the entire sample, with no significant difference in the physician-patient rapport among those who provided video or audio consultations compared to face-to-face consultations (*P*=.95) (Table 3). Interestingly, when compared to face-to-face consultations, the proportion of physicians who perceived that telemedicine reduces the overall documentation time (*P*<.001) and increases the total number of patient consultations (*P*=.01) was significantly higher among physicians who provided audio consultations than among those who provided video consultations. The proportion of female physicians who agreed that telemedicine decreases the overall documentation time and increases the total number of patient consultations was substantially higher than that of their male counterparts (*P*=.008 and *P*<.001, respectively) (Multimedia Appendix 1).

**Table 3:** Comparison of survey responses on perceived professional productivity by modality.

Perceived professional productivity			Audio consultations, n (%)	Video consultations, n (%)		Total, n (%)		*P* value	
**Patient’s rapport rather than face-to-face consultations**									.95
	Disagree and strongly disagree	228 (65.71)		179 (64.86)	407 (65.33)				
	Neutral	83 (23.92)		69 (25.00)	152 (24.40)				
	Agree and strongly agree	36 (10.37)		28 (10.14)	64 (10.27)				
**Reduced overall consultation time rather than face-to-face consultations**									0.066
	Disagree and strongly disagree	80 (23.05)		84 (30.43)	164 (26.32)				
	Neutral	94 (27.09)		77 (27.9)	171 (27.45)				
	Agree and strongly agree	173(49.86		115 (41.67)	288 (46.23)				
**Reduced overall documentation time rather than face-to-face consultations**									<0.001
	Disagree and strongly disagree	84 (24.21)		104 (37.68)	188 (30.18)				
	Neutral	77 (22.19)		77 (27.9)	154 (24.72)				
	Agree and strongly agree	186 (53.6)		95 (34.42)	281 (45.1)				
**Increased total number of consulted patients rather than face-to-face consultations**									0.01
	Disagree and strongly disagree	89 (25.65)		95 (34.42)	184 (29.53)				
	Neutral	112 (32.28)		94 (34.06)	206 (33.07)				
	Agree and strongly agree	146 (42.07)		87 (31.52)	233 (37.4)				

### Working Experience, Satisfaction, and Barriers to Telemedicine

The majority of physicians who provided video consultations agreed that they received sufficient technological support during the web-based consultation; this proportion was greater than that of physicians who provided audio consultations (76.45% vs 53.60%, respectively; *P*<.001).

There was no significant difference in the satisfaction with the quality of the clinical consultation between physicians who provided video consultations and those who provided audio consultations (*P*=.07).

On assessing the barriers to telemedicine, physicians who provided audio consultations reported that the “inability to see the patient during the consultation” was a significant barrier to the quality of the remote clinical consultations (*P*=.001), and they preferred not to use telemedicine services owing to low payment and reimbursement rates (*P*=.004), were unable to confirm the patient’s identity during the audio consultation (*P*=.04), and reported that lack of training is a barrier to the use of telemedicine services to provide remote care to patients (*P*<.001) (Multimedia Appendix 1).

### Multivariate Analysis

In the multivariate regression model, video consultations were associated with significantly improved confidence toward the management of acute conditions (OR 1.62, 95% CI 1.2-2.21; *P*=.002) and increased perceived ability to provide patient education (OR 2.21, 95% CI 1.04-4.33; *P*=.04), while male sex was associated with a lower perceived ability to provide patient education during the web-based consultation (OR 0.48, 95% CI 0.27-0.84; *P*=.01). There was no significant difference in physician’s confidence in managing chronic conditions or conducting follow-up consultations among those who provided audio or video consultations Table 4. Additionally, previous experience with frequent telemedicine consultations was significantly associated with higher confidence in diagnosing acute conditions (OR=2.12, 95% CI:1.04-4.33 *P*=.039) and with a lower perceived risk of misdiagnosis (OR 0.46, 95% CI 0.31-0.68; *P*<.001). Our analysis also shows that video consultations were significantly associated with a perceived increase in overall consultation time, overall documentation time, and a reduction in the overall number of patients consulted when compared to face-to-face clinical consultations. Previous experience with telemedicine was significantly associated with the perception of an enhanced physician-patient rapport and the perception of an increased total number of patient consultations when compared to face-to-face consultations (Table 5).

**Table 4:** Adjusted multivariate analysis for the perceived quality of clinical consultations.

Variables			Confidence in managing acute conditions				Confidence in managing chronic conditions and follow-up consultations				Ability to answer patient questions				Ability to provide patient health education				Perceived risk of misdiagnosis with telemedicine		
			OR^a^ (95% CI)		*P* value		OR (95% CI)		*P* value		OR (95% CI)		*P* value		OR (95% CI)		*P* value		OR (95% CI)		*P* value
Modality (video vs audio consultations)			1.62 (1.2-2.21)		.002		1.35 (0.88-2.08)		.17		1.6 (0.94-2.74)		.08		2.02 (1.19-3.41)		.009		0.81 (0.58-1.12)		.20
Sex (male vs female)			0.84 (0.61-1.16)		.29		0.76 (0.48-1.20)		.24		0.57 (0.32-1.02)		.06		0.48 (0.27-0.84)		.01		1.23 (0.87-1.74)		.25
**Age (years)**																					
	40-49 vs <39	0.9 (0.6-1.36)		.62		1.16 (0.66-2.04)		.61		1.06 (0.52-2.13)		.88		1.66 (0.85-3.25)		.14		1.23 (0.8-1.89)		.35	
	50-59 vs <39	0.95 (0.6-1.51)		.83		1.53 (0.79-2.94)		.20		1.49 (0.67-3.33)		.33		1.34 (0.65-2.76)		.44		1.11 (0.67-1.82)		.68	
	≥60 vs <39	0.82 (0.46-1.44)		.49		1.7 (0.74-3.94)		.21		1.11 (0.43-2.92)		.83		2.17 (0.81-5.82)		.13		0.85 (0.47-1.54)		.60	
**Specialty**																					
	Surgical specialties vs internal medicine	1.42 (0.83-2.41)		.20		1.06 (0.51-2.20)		.87		0.95 (0.41-2.19)		.90		1.42 (0.59-3.37)		.43		1.11 (0.62-1.99)		.71	
	Family medicine vs internal medicine	1.46 (0.92-2.32)		.11		1.38 (0.69-2.74)		.36		1.56 (0.61-3.95)		.35		1.26 (0.56-2.87)		.58		0.67 (0.42-1.09)		.10	
	Others vs internal medicine	1.52 (0.79-2.94)		.21		0.18 (0.09-0.37)		<.001		0.21 (0.09-0.49)		<.001		0.36 (0.16-0.84)		.02		0.54 (0.29-1.03)		.06	
**Physician designation**																					
	Resident vs general physician	1.03 (0.33-3.19)		.96		0.74 (0.16-3.34)		.70		0.30 (0.06-1.47)		.14		0.66 (0.11-3.77)		.64		1.00 (0.31-3.23)		.99	
	Specialist vs general physician	1.34 (0.81-2.20)		.26		0.93 (0.46 -1.87)		.84		0.70 (0.28-1.74)		.45		0.67 (0.29-1.57)		.36		1.20 (0.72-2.00)		.48	
	Consultant vs general physician	0.99 (0.56-1.75)		.96		0.48 (0.22-1.06)		.07		0.49 (0.17-1.36)		.17		0.57 (0.21-1.51)		.26		1.18 (0.65-2.15)		.58	
**Past experience with telemedicine**																					
	Used few times vs never used	1.31 (0.88-1.93)		.18		0.79 (0.48-1.33)		.38		1.36 (0.69-2.70)		.38		1.43 (0.74-2.77)		.29		0.46 (0.31-0.68)		<.001	
	Used frequently vs never used	2.12 (1.04-4.33)		.04		1.37 (0.46-4.10)		.57		3.65 (0.48-27.63)		.21		1.26 (0.36-4.41)		.71		0.45 (0.22-0.91)		.03	

^a^OR: odds ratio.

**Table 5:** Adjusted multivariate analysis for perceived professional productivity.

Variables		Patient rapport rather than face-to-face consultations		Reduced overall consultation time rather than face-to-face consultations		Reduced overall documentation time rather than face-to-face consultations		Total number of consulted patients rather than face-to-face consultations	
		OR^a^ (95% CI)	*P* value	OR (95% CI)	*P* value	OR (95% CI)	*P* value	OR (95% CI)	*P* value
Modality (video vs audio consultation		1.07 (0.76-1.49)	.71	0.69 (0.51-0.93)	.02	0.48 (0.36-0.65)	<.001	0.66 (0.49-0.89)	.006
Sex (male vs female)		0.69 (0.48-0.99)	.04	1.00 (0.73-1.39)	.98	0.72 (0.52-1.00)	.05	0.60 (0.43-0.82)	.002
**Age (years)**									
	40-49 vs <39	1.00 (0.64-1.56)	.99	0.75 (0.50-1.13)	.17	0.80 (0.53-1.20)	.28	0.81 (0.54-1.22)	.32
	50-59 vs <39	1.40 (0.84-2.32)	.20	1.15 (0.72-1.84)	.56	0.89 (0.56-1.43)	.64	1.10 (0.70-1.76)	.67
	≥60 vs <39	1.31 (0.70-2.44)	.39	1.25 (0.70-2.21)	.45	1.40 (0.78-2.53)	.26	1.26 (0.72-2.21)	.42
**Specialty**									
	Surgical specialties vs internal medicine	2.03 (1.15-3.59)	.02	1.08 (0.64-1.81)	.78	0.94 (0.56-1.59)	.83	1.20 (0.73-1.97)	.48
	Family medicine vs internal medicine	0.93 (0.56-1.53)	.77	1.30 (0.83-2.03)	.26	1.34 (0.84-2.15)	.22	1.19 (0.75-1.89)	.46
	Others vs internal medicine	1.45 (0.74-2.84)	.27	0.74 (0.41-1.36)	.34	0.78 (0.42-1.44)	.43	1.22 (0.65-2.28)	.54
**Physician designation**									
	Resident vs general physician	1.45 (0.46-4.63)	.53	0.97 (0.33-2.88)	.96	0.99 (0.3-3.25)	.98	0.76 (0.23-2.51)	.65
	Specialist vs general physician	0.85 (0.51-1.43)	.54	0.99 (0.61-1.60)	.97	1.11 (0.67-1.82)	.69	0.91 (0.56-1.48)	.70
	Consultant vs general physician	0.46 (0.25-0.86)	.02	0.61 (0.35-1.05)	.08	0.66 (0.37-1.17)	.16	0.59 (0.33-1.03)	.06
**Past experience with telemedicine**									
	Used few times vs never used	1.46 (0.96-2.23)	.08	0.96 (0.66-1.41)	.85	0.96 (0.65-1.41)	.84	1.46 (0.99-2.14)	.05
	Used frequently vs never used	2.49 (1.26-4.90)	.008	1.72 (0.84-3.54)	.14	0.80 (0.41-1.57)	.52	2.81 (1.38-5.71)	.004

^a^OR: odds ratio.

## Discussion

### Principal Findings

This analysis of 623 physicians shows that video consultations are independently associated with a 62% increase in confidence in managing acute conditions, and physicians who provided video consultations were 2-fold more likely to provide patient education during the web-based consultations. Moreover, previous experience with telemedicine was associated with a 2-fold increase in confidence in managing acute conditions and a 55% reduction in the perception of the risk of misdiagnosis. More than one-third (37.68%) of physicians who provided video consultations did not agree that telemedicine reduces the overall consultation time, and approximately one-third (34.42%) did not agree that telemedicine increases the overall number of patient consultations when compared to face-to-face visits. Additionally, those who had previous experience with telemedicine were 2.5-fold more likely to build a rapport with their patients and 2.8-fold more likely to perceive that telemedicine increases the total number of patient consultations when compared to face-to-face consultations.

The COVID-19 pandemic provided sufficient incentive for the health care system to shift to web-based care to minimize the exposure to SARS-CoV-2 [19,33] and ultimately, as reported by Portnoy et al, “the only virus one can get while doing telemedicine is a cyber virus” [34,35]. The presence of these different modalities of telemedicine provided different opportunities for patients to connect with their health care providers, with rapid implementation of video and audio consultations partially owing to the availability of smartphones and the ubiquity of videoconferencing apps, since cameras are now an essential feature of these cellphones [36-42].

Although data on physician experience and outcome quality with each modality are limited, our first key finding suggests that when evaluating a patient for the first time or a patient with an acute condition, there is an added value in using videoconferencing apps to evaluate the patient’s general state of health, which is pivotal to the clinical decision-making process [43,44]. Because medical presentations can vary in acuity and thus warrant different management approaches, physicians may need a real-time modality to assess the patient better, view the site of pathology, discuss treatment options, address the patient’s concerns, and promote compliance with the treatment regimen. Video consultations can proximate real-life visits to a great extent as both the physician and patient can interact with each other simultaneously; this negates the psychological distance by allowing facial expressions and body language to be observed and interpreted, thus promoting empathic communication and the generation of a physician-patient rapport [45]. Therefore, a video consultation may be preferable when consulting a new patient for the first time as physicians would feel more confident in making diagnostic and treatment decisions. However, when evaluating follow-up patients with chronic diseases or for medication refill, video and audio telemedicine may be of equal quality and have similar outcomes as reported here and in previous studies [35,46-48]. These results may also help policymakers in low- and middle-income countries in applying reasonable protocols for selecting either video or audio consultations for patients who live in geographically remote areas or those who require frequent follow-up evaluation [49]. For instance, video consultations could be used for new or mild-to-moderate clinical presentations where real-time evaluation is needed, while audio consultations could be reserved for follow-up patients with chronic medical conditions or those with nonurgent medical problems who need to travel long distances and incur out-of-pocket costs [50]. In this course, a double triage system may be needed where a triage nurse consults with the patient who requests a telemedicine appointment and assess the patient’s triage level using the Triage and Acuity Scale before recommending an in-person visit or video or audio consultation for the patient [51].

Our second key finding is that previous experience with telemedicine was associated with a lower perceived risk of misdiagnosis. In this respect, the more physicians were trained on telemedicine, the more confident they were in making a clinical diagnosis and the lesser the impression of a medical malpractice they had. Our results emphasize the need to increase telemedicine competencies in residency training and other clinical programs. For example, it is important to provide a formal education on best practices on how to remotely assess a patient’s chief complaint and vital signs and carry out remote physical examination before placing physicians in web-based clinics, as prior experience with telemedicine can increase readiness and preparedness to carry out web-based consultations. This is intuitive specially for physicians who frequently use telemedicine, including those involved in internal medicine and family medicine [52]. Our findings are consistent with those of previous studies [53-55]. Ha et al reported that physicians who had a structured educational program in telemedicine had greater confidence in addressing clinical problems than those who did not receive an educational program [56]. Furthermore, Moore et al reported that the lack of telemedicine training was a barrier to provide telemedicine services among family medicine residents [52].

Our third key finding is that video consultations were associated with a perceived increase in overall consultation time, increased documentation time, and decreased total number of patient consultations. It is plausible that video consultations lasted longer owing to several reasons including technical difficulties related to internet connection, poor audio or speaker quality, disruption to the conversation flow, and difficulties with guiding a remote physical examination. In face-to-face interactions, people see and hear each other’s words as they are produced; however, when using videoconferencing platforms, actions and words are heard milliseconds later. These delays, although small, are meaningful and can interfere with the conversation flow and result in miscommunication, thus consuming more time in an attempt to understand patients’ problems and physicians’ instructions [14,57,58]. Moreover, during video consultations, the physician may guide the patient through remote physical examination, which may increase the duration of the clinical consultation. Subsequently, the total number of daily patient consultations is expected to decrease owing to an increased duration of consultations in a limited clinical schedule.

Our fourth key finding is the identification of elements that represent barriers to telemedicine. A physician’s inability to see the patient during the remote consultation could restrict tele-examination of the patient, where a guided remote assessment of the underlying condition is not feasible owing due to limited interaction with the web-based interface and the patient’s difficulty to follow clinical instructions without physically seeing the provider’s technique [59]. Moreover, the inability to see the patient during the clinical consultation could raise serious security and privacy issues, since the physician may not be able to confirm the patient’s identity during the remote consultation, thus emphasizing the need of guidelines on identity management and security considerations to protect the patient’s privacy during both audio and video consultations. Additionally, reimbursement issues with audio and video consultations need to be acknowledged, as it does not appear to attract health care providers preferentially for the delivery of telecare services. The current payment plans have been confusing, as the telemedicine provider needs to consider different private and governmental insurance policies when providing a remote consultation [60]. This confusion has been also a major deterrent to the use of telemedicine services. Furthermore, the relative difference in cost between telemedicine visits and a comparable face-to-face visit has been one of the barriers to the use of telemedicine. If a telemedicine visit is remunerated at a lower value than an equivalent face-to-face visit, physicians would be less willing to increase the provision of this service. There is a need to establish standardized regulations and billing rules to control costs. In principle, reimbursement costs for teleconsultation need to be equivalent to those of face-to-face visits to increase the adoption of telemedicine services [60]. A lack of training on how to treat a patient remotely may also be an obstacle that jeopardizes the efficiency of the virtual consultations, which must be overcome by incorporating appropriate training curricula, which can be incorporated through physician training programs.

### Limitations and Strengths

This study has several limitations that we intend to address in future studies. First, this was an observational study that reflects outcomes with video and audio telemedicine consultations at a single point in time. Second, data on what reimbursement challenges are associated with each modality was not captured in detail in this study, which might have biased physicians’ attitudes toward each modality. Third, the perception of misdiagnosis was not defined in our survey; hence, it was challenging to understand the association between this outcome and predictors for physicians who used video or audio consultation. Fourth, in this study, patients’ preferences for video or audio consultations were not captured and thus could have affected the number of clinical consultations for each modality and might have biased physicians’ attitudes toward the mode of remote consultation.

Despite these limitations, our study has several strengths. To our knowledge, this study was one of the first comprehensive telemedicine studies in the Middle Eastern region, which had a nationally representative sample of physicians who used telemedicine and had a high survey response rate. Additionally, our study measured the difference in physicians’ attitudes toward telemedicine by modality type, which is informative for policymaking decisions.

### Conclusions

The experience with the COVID-19 pandemic has highlighted the important role of telemedicine in emergency responses. While we may not be able to precisely predict the exact diagnostic outcomes with each telemedicine modality, there is, however, a growing body of evidence that suggests that video consultations are associated with improved physician confidence in managing acute conditions and a greater ability to provide patient education during web-based consultations. This study demonstrates that when managing chronic conditions or follow-up patients remotely, audio consultation is as suitable as video consultation to health care providers. These findings may be helpful for health care policymakers in low-to-middle–income countries to provide ample health care access to patients with chronic and noncommunicable diseases. Previous experience with telemedicine was associated with improved physicians’ confidence in case management, a lower perceived risk of misdiagnosis, an increased ability to provide patients with health education, and a better physician-patient rapport. Telemedicine services are likely to be retained, and as we build our telehealth system, it is intuitive to prioritize the “new normal” and implement a structured telemedicine curriculum in physician training programs and prepare them for web-based consultations. It is also necessary to acknowledge the barriers to telemedicine and create solutions and regulations to overcome these obstacles and increase the service adoption rate.

## Appendix

Multimedia Appendix 1 Supplementary tables with additional results.

## Abbreviations

OR: odds ratio
VIF: variance inflation factor
