# Supplementary material for: Physicians’ Attitudes Toward Telemedicine Consultations During the COVID-19 Pandemic: Cross-sectional Study
Source: JMIR Med Inform. 2021 Jun 1;9(6):e29251. doi: 10.2196/29251 (PMC8171285; doi:10.2196/29251)
Supplement: Multimedia Appendix 1 [file medinform_v9i6e29251_app1.docx]

**Supplement**

Table 1 Comparison of survey responses on perceived quality of clinical care provided by sex

| **Perceived Quality of Clinical Care Provided** | **Female** | **Male** | **Total** | ***P* Value** |
| --- | --- | --- | --- | --- |
|  | **n (%) 270 (43.3)** | **n (%) 353 (56.7)** | **n (%)**  **623 (100)** |  |
| **Confidence in managing acute consultations** |  |  |  | 0.046 |
| Disagree & Strongly Disagree | 60 (22.22) | 72 (20.4) | 132 (21.19) |  |
| Neutral | 75 (27.78) | 131 (37.11) | 206 (33.07) |  |
| Agree & Strongly Agree | 135 (50) | 150 (42.49) | 285 (45.75) |  |
| **Confidence in managing chronic and follow-up consultations** |  |  |  | 0.561 |
| Disagree & Strongly Disagree | 11 (4.07) | 15 (4.25) | 26 (4.17) |  |
| Neutral | 34 (12.59) | 55 (15.58) | 89 (14.29) |  |
| Agree & Strongly Agree | 225 (83.33) | 283 (80.17) | 508 (81.54) |  |
| **Ability to answer patient's questions** |  |  |  | 0.121 |
| Disagree & Strongly Disagree | 3 (1.11) | 9 (2.55) | 12 (1.93) |  |
| Neutral | 20 (7.41) | 39 (11.05) | 59 (9.47) |  |
| Agree & Strongly Agree | 247 (91.48) | 305 (86.4) | 552 (88.6) |  |
| **Ability to provide patient health education** |  |  |  | 0.022 |
| Disagree & Strongly Disagree | 8 (2.96) | 8 (2.27) | 16 (2.57) |  |
| Neutral | 16 (5.93) | 44 (12.46) | 60 (9.63) |  |
| Agree & Strongly Agree | 246 (91.11) | 301 (85.27) | 547 (87.8) |  |
| **Perceived risk of misdiagnosis with Telemedicine** |  |  |  | 0.021 |
| Disagree & Strongly Disagree | 47 (17.41) | 35 (9.92) | 82 (13.16) |  |
| Neutral | 65 (24.07) | 99 (28.05) | 164 (26.32) |  |
| Agree & Strongly Agree | 158 (58.52) | 219 (62.04) | 377 (60.51) |  |

Table 2 Comparison of survey responses on perceived quality of clinical care provided by age

| **Perceived Quality of Clinical Care Provided** | <39 | 40-49 | 50-59 | 60+ | Total | ***P* Value** |
| --- | --- | --- | --- | --- | --- | --- |
|  | **n (%) 146 (23.43)** | **n (%) 254 (40.77)** | **n (%) 145 (23.27)** | **n (%) 78 (12.52)** | **n (%)**  **623 (100)** |  |
| **Confidence in managing acute consultations** |  |  |  |  |  | 0.474 |
| Disagree & Strongly Disagree | 29 (19.86) | 51 (20.08) | 29 (20) | 23 (29.49) | 132 (21.19) |  |
| Neutral | 44 (30.14) | 90 (35.43) | 51 (35.17) | 21 (26.92) | 206 (33.07) |  |
| Agree & Strongly Agree | 73 (50) | 113 (44.49) | 65 (44.83) | 34 (43.59) | 285 (45.75) |  |
| **Confidence in managing chronic and follow-up consultations** |  |  |  |  |  | 0.414 |
| Disagree & Strongly Disagree | 8 (5.48) | 7 (2.76) | 6 (4.14) | 5 (6.41) | 26 (4.17) |  |
| Neutral | 23 (15.75) | 40 (15.75) | 20 (13.79) | 6 (7.69) | 89 (14.29) |  |
| Agree & Strongly Agree | 115 (78.77) | 207 (81.5) | 119 (82.07) | 67 (85.9) | 508 (81.54) |  |
| **Ability to answer patient's questions** |  |  |  |  |  | 0.802 |
| Disagree & Strongly Disagree | 3 (2.05) | 5 (1.97) | 1 (0.69) | 3 (3.85) | 12 (1.93) |  |
| Neutral | 15 (10.27) | 24 (9.45) | 14 (9.66) | 6 (7.69) | 59 (9.47) |  |
| Agree & Strongly Agree | 128 (87.67) | 225 (88.58) | 130 (89.66) | 69 (88.46) | 552 (88.6) |  |
| **Ability to provide patient health education** |  |  |  |  |  | 0.136 |
| Disagree & Strongly Disagree | 8 (5.48) | 4 (1.57) | 2 (1.38) | 2 (2.56) | 16 (2.57) |  |
| Neutral | 13 (8.9) | 23 (9.06) | 19 (13.1) | 5 (6.41) | 60 (9.63) |  |
| Agree & Strongly Agree | 125 (85.62) | 227 (89.37) | 124 (85.52) | 71 (91.03) | 547 (87.8) |  |
| **Perceived risk of misdiagnosis with Telemedicine** |  |  |  |  |  | 0.307 |
| Disagree & Strongly Disagree | 20 (13.7) | 30 (11.81) | 18 (12.41) | 14 (17.95) | 82 (13.16) |  |
| Neutral | 48 (32.88) | 60 (23.62) | 38 (26.21) | 18 (23.08) | 164 (26.32) |  |
| Agree & Strongly Agree | 78 (53.42) | 164 (64.57) | 89 (61.38) | 46 (58.97) | 377 (60.51) |  |

Table 3 Comparison of survey responses on perceived quality of clinical care provided by physician rank

| **Perceived Quality of Clinical Care Provided** | GP | Resident | Specialist | Consultant | Total | ***P* Value** |
| --- | --- | --- | --- | --- | --- | --- |
|  | **n (%)**  **103 (16.53)** | **n (%)**  **11 (1.77)** | **n (%)**  **364 (58.43)** | **n (%)**  **145 (23.27)** | **n (%)**  **623 (100)** |  |
| **Confidence in managing acute consultations** |  |  |  |  |  | 0.21 |
| Disagree & Strongly Disagree | 25 (24.27) | 1 (9.09) | 68 (18.68) | 38 (26.21) | 132 (21.19) |  |
| Neutral | 28 (27.18) | 6 (54.55) | 123 (33.79) | 49 (33.79) | 206 (33.07) |  |
| Agree & Strongly Agree | 50 (48.54) | 4 (36.36) | 173 (47.53) | 58 (40) | 285 (45.75) |  |
| **Confidence in managing chronic and follow-up consultations** |  |  |  |  |  | 0.347 |
| Disagree & Strongly Disagree | 2 (1.94) | 1 (9.09) | 13 (3.57) | 10 (6.9) | 26 (4.17) |  |
| Neutral | 16 (15.53) | 2 (18.18) | 47 (12.91) | 24 (16.55) | 89 (14.29) |  |
| Agree & Strongly Agree | 85 (82.52) | 8 (72.73) | 304 (83.52) | 111 (76.55) | 508 (81.54) |  |
| **Ability to answer patient's questions** |  |  |  |  |  | 0.316 |
| Disagree & Strongly Disagree | 1 (0.97) | - | 6 (1.65) | 5 (3.45) | 12 (1.93) |  |
| Neutral | 8 (7.77) | 3 (27.27) | 34 (9.34) | 14 (9.66) | 59 (9.47) |  |
| Agree & Strongly Agree | 94 (91.26) | 8 (72.73) | 324 (89.01) | 126 (86.9) | 552 (88.6) |  |
| **Ability to provide patient health education** |  |  |  |  |  | 0.895 |
| Disagree & Strongly Disagree | 2 (1.94) | - | 9 (2.47) | 5 (3.45) | 16 (2.57) |  |
| Neutral | 8 (7.77) | 2 (18.18) | 36 (9.89) | 14 (9.66) | 60 (9.63) |  |
| Agree & Strongly Agree | 93 (90.29) | 9 (81.82) | 319 (87.64) | 126 (86.9) | 547 (87.8) |  |
| **Perceived risk of misdiagnosis with Telemedicine** |  |  |  |  |  | 0.236 |
| Disagree & Strongly Disagree | 20 (19.42) | 1 (9.09) | 41 (11.26) | 20 (13.79) | 82 (13.16) |  |
| Neutral | 29 (28.16) | 5 (45.45) | 95 (26.1) | 35 (24.14) | 164 (26.32) |  |
| Agree & Strongly Agree | 54 (52.43) | 5 (45.45) | 228 (62.64) | 90 (62.07) | 377 (60.51) |  |

Table 4 Comparison of survey responses on perceived professional productivity by sex

| **Perceived Professional Productivity** | **Female** | **Male** | **Total** | ***P* Value** |
| --- | --- | --- | --- | --- |
|  | **n (%) 270 (43.3)** | **n (%) 353 (56.7)** | **n (%)**  **623 (100)** |  |
| **Patient's rapport more than face-to-face visits** |  |  |  | 0.277 |
| Disagree & Strongly Disagree | 167 (61.85) | 240 (67.99) | 407 (65.33) |  |
| Neutral | 73 (27.04) | 79 (22.38) | 152 (24.4) |  |
| Agree & Strongly Agree | 30 (11.11) | 34 (9.63) | 64 (10.27) |  |
| **Reduced overall consultation time more than face-to-face visits** |  |  |  | 0.418 |
| Disagree & Strongly Disagree | 65 (24.07) | 99 (28.05) | 164 (26.32) |  |
| Neutral | 80 (29.63) | 91 (25.78) | 171 (27.45) |  |
| Agree & Strongly Agree | 125 (46.3) | 163 (46.18) | 288 (46.23) |  |
| **Reduced overall documentation time more than face-to-face visits** |  |  |  | 0.008 |
| Disagree & Strongly Disagree | 71 (26.30) | 117 (33.14) | 188 (30.18) |  |
| Neutral | 58 (21.48) | 96 (27.2) | 154 (24.72) |  |
| Agree & Strongly Agree | 141 (52.22) | 140 (39.66) | 281 (45.1) |  |
| **Increased total number of consulted patients more than face-to-face visits** |  |  |  | <0.001 |
| Disagree & Strongly Disagree | 67 (24.81) | 117 (33.14) | 184 (29.53) |  |
| Neutral | 78 (28.89) | 128 (36.26) | 206 (33.07) |  |
| Agree & Strongly Agree | 125 (46.3) | 108 (30.59) | 233 (37.4) |  |

Table 5 Comparison of survey responses on perceived professional productivity by age

| **Perceived Professional Productivity** | <39 | 40-49 | 50-59 | 60+ | Total | ***P* Value** |
| --- | --- | --- | --- | --- | --- | --- |
|  | **n (%) 146 (23.43)** | **n (%) 254 (40.77)** | **n (%) 145 (23.27)** | **n (%) 78 (12.52)** | **n (%)**  **623 (100)** |  |
| **Patient's rapport more than face-to-face visits** |  |  |  |  |  | 0.235 |
| Disagree & Strongly Disagree | 91 (62.33) | 172 (67.72) | 92 (63.45) | 52 (66.67) | 407 (65.33) |  |
| Neutral | 44 (30.14) | 58 (22.83) | 31 (21.38) | 19 (24.36) | 152 (24.4) |  |
| Agree & Strongly Agree | 11 (7.53) | 24 (9.45) | 22 (15.17) | 7 (8.97) | 64 (10.27) |  |
| **Reduced overall consultation time more than face-to-face visits** |  |  |  |  |  | 0.305 |
| Disagree & Strongly Disagree | 32 (21.92) | 76 (29.92) | 36 (24.83) | 20 (25.64) | 164 (26.32) |  |
| Neutral | 42 (28.77) | 75 (29.53) | 37 (25.52) | 17 (21.79) | 171 (27.45) |  |
| Agree & Strongly Agree | 72 (49.32) | 103 (40.55) | 72 (49.66) | 41 (52.56) | 288 (46.23) |  |
| **Reduced overall documentation time more than face-to-face visits** |  |  |  |  |  | 0.118 |
| Disagree & Strongly Disagree | 35 (23.97) | 81 (31.89) | 47 (32.41) | 25 (32.05) | 188 (30.18) |  |
| Neutral | 37 (25.34) | 68 (26.77) | 38 (26.21) | 11 (14.1) | 154 (24.72) |  |
| Agree & Strongly Agree | 74 (50.68) | 105 (41.34) | 60 (41.38) | 42 (53.85) | 281 (45.1) |  |
| **Increased total number of consulted patients more than face-to-face visits** |  |  |  |  |  | 0.27 |
| Disagree & Strongly Disagree | 39 (26.71) | 80 (31.5) | 42 (28.97) | 23 (29.49) | 184 (29.53) |  |
| Neutral | 42 (28.77) | 94 (37.01) | 45 (31.03) | 25 (32.05) | 206 (33.07) |  |
| Agree & Strongly Agree | 65 (44.52) | 80 (31.5) | 58 (40) | 30 (38.46) | 233 (37.4) |  |

Table 6 Comparison of survey responses on perceived professional productivity by physician rank

| **Perceived Professional Productivity** | GP | Resident | Specialist | Consultant | Total | ***P* Value** |
| --- | --- | --- | --- | --- | --- | --- |
|  | **n (%)**  **103 (16.53)** | **n (%)**  **11 (1.77)** | **n (%)**  **364 (58.43)** | **n (%)**  **145 (23.27)** | **n (%)**  **623 (100)** |  |
| **Patient's rapport more than face-to-face visits** |  |  |  |  |  | 0.031 |
| Disagree & Strongly Disagree | 61 (59.22) | 5 (45.45) | 235 (64.56) | 106 (73.1) | 407 (65.33) |  |
| Neutral | 34 (33.01) | 5 (45.45) | 83 (22.8) | 30 (20.69) | 152 (24.4) |  |
| Agree & Strongly Agree | 8 (7.77) | 1 (9.09) | 46 (12.64) | 9 (6.21) | 64 (10.27) |  |
| **Reduced overall consultation time more than face-to-face visits** |  |  |  |  |  | 0.007 |
| Disagree & Strongly Disagree | 19 (18.45) | - | 97 (26.65) | 48 (33.1) | 164 (26.32) |  |
| Neutral | 34 (33.01) | 7 (63.64) | 90 (24.73) | 40 (27.59) | 171 (27.45) |  |
| Agree & Strongly Agree | 50 (48.54) | 4 (36.36) | 177 (48.63) | 57 (39.31) | 288 (46.23) |  |
| **Reduced overall documentation time more than face-to-face visits** |  |  |  |  |  | 0.038 |
| Disagree & Strongly Disagree | 22 (21.36) | 2 (18.18) | 105 (28.85) | 59 (40.69) | 188 (30.18) |  |
| Neutral | 31 (30.1) | 4 (36.36) | 90 (24.73) | 29 (20) | 154 (24.72) |  |
| Agree & Strongly Agree | 50 (48.54) | 5 (45.45) | 169 (46.43) | 57 (39.31) | 281 (45.1) |  |
| **Increased total number of consulted patients more than face-to-face visits** |  |  |  |  |  | 0.326 |
| Disagree & Strongly Disagree | 22 (21.36) | 3 (27.27) | 108 (29.67) | 51 (35.17) | 184 (29.53) |  |
| Neutral | 35 (33.98) | 3 (27.27) | 120 (32.97) | 48 (33.1) | 206 (33.07) |  |
| Agree & Strongly Agree | 46 (44.66) | 5 (45.45) | 136 (37.36) | 46 (31.72) | 233 (37.4) |  |

Table 7 Comparison of survey responses on telemedicine working experience by modality

| **Telemedicine Working Experience** | **Audio Consultation** | **Video Consultation** | **Total** | ***P* Value** |
| --- | --- | --- | --- | --- |
|  | **n (%) 347 (55.7)** | **n (%) 276 (44.3)** | **n (%)**  **623 (100)** |  |
| **Sufficient technological support during the implementation process** |  |  |  | <0.001 |
| Disagree & Strongly Disagree | 47 (13.54) | 24 (8.7) | 71 (11.4) |  |
| Neutral | 114 (32.85) | 41 (14.86) | 155 (24.88) |  |
| Agree & Strongly Agree | 186 (53.6) | 211 (76.45) | 397 (63.72) |  |
| **Convenient for clinical practice during the pandemic** |  |  |  | 0.272 |
| Disagree & Strongly Disagree | 37 (10.66) | 22 (7.97) | 59 (9.47) |  |
| Neutral | 76 (21.9) | 52 (18.84) | 128 (20.55) |  |
| Agree & Strongly Agree | 234 (67.44) | 202 (73.19) | 436 (69.98) |  |
| **Promoted patient's adherence to appointment time more than office visits** |  |  |  | 0.297 |
| Disagree & Strongly Disagree | 103 (29.68) | 76 (27.54) | 179 (28.73) |  |
| Neutral | 113 (32.56) | 79 (28.62) | 192 (30.82) |  |
| Agree & Strongly Agree | 131 (37.75) | 121 (43.84) | 252 (40.45) |  |

Table 8 Comparison of survey responses on Satisfaction with Telemedicine by modality

| **Satisfaction with Telemedicine** | **Audio Consultation** | **Video Consultation** | **Total** | ***P* Value** |
| --- | --- | --- | --- | --- |
|  | **n (%) 347 (55.7)** | **n (%) 276 (44.3)** | **n (%)**  **623 (100)** |  |
| **Improved access to healthcare services** |  |  |  | 0.146 |
| Disagree & Strongly Disagree | 19 (5.48) | 8 (2.9) | 27 (4.33) |  |
| Neutral | 46 (13.26) | 29 (10.51) | 75 (12.04) |  |
| Agree & Strongly Agree | 282 (81.27) | 239 (86.59) | 521 (83.63) |  |
| **Improved patient’s health status** |  |  |  | 0.065 |
| Disagree & Strongly Disagree | 51 (14.7) | 28 (10.14) | 79 (12.68) |  |
| Neutral | 100 (28.82) | 68 (24.64) | 168 (26.97) |  |
| Agree & Strongly Agree | 196 (56.48) | 180 (65.22) | 376 (60.35) |  |
| **Satisfied with telemedicine services** |  |  |  | 0.066 |
| Disagree & Strongly Disagree | 27 (7.78) | 13 (4.71) | 40 (6.42) |  |
| Neutral | 92 (26.51) | 59 (21.38) | 151 (24.24) |  |
| Agree & Strongly Agree | 228 (65.71) | 204 (73.91) | 432 (69.34) |  |
| **Support the use of telemedicine after the pandemic for certain patients** |  |  |  | 0.248 |
| Disagree & Strongly Disagree | 58 (16.71) | 36 (13.04) | 94 (15.09) |  |
| Neutral | 44 (12.68) | 45 (16.3) | 89 (14.29) |  |
| Agree & Strongly Agree | 245 (70.61) | 195 (70.65) | 440 (70.63) |  |

Table 9 Comparison of survey responses on perceived barriers to telemedicine by modality

| **Perceived Barriers to Telemedicine** | **Audio Consultation** | **Video Consultation** | **Total** | ***P* Value** |
| --- | --- | --- | --- | --- |
|  | **n (%) 347 (55.7)** | **n (%) 276 (44.3)** | **n (%)**  **623 (100)** |  |
| **Inability to see the patient** |  |  |  | <0.001 |
| Disagree & Strongly Disagree | 27 (7.78) | 36 (13.04) | 63 (10.11) |  |
| Neutral | 58 (16.71) | 72 (26.09) | 130 (20.87) |  |
| Agree & Strongly Agree | 262 (75.5) | 168 (60.87) | 430 (69.02) |  |
| **Challenges with Insurance coverage** |  |  |  | 0.004 |
| Disagree & Strongly Disagree | 55 (15.85) | 73 (26.45) | 128 (20.55) |  |
| Neutral | 195 (56.2) | 129 (46.74) | 324 (52.01) |  |
| Agree & Strongly Agree | 97 (27.95) | 74 (26.81) | 171 (27.45) |  |
| **Patients discomfort during the remote consultation** |  |  |  | 0.163 |
| Disagree & Strongly Disagree | 148 (42.65) | 97 (35.14) | 245 (39.33) |  |
| Neutral | 130 (37.46) | 117 (42.39) | 247 (39.65) |  |
| Agree & Strongly Agree | 69 (19.88) | 62 (22.46) | 131 (21.03) |  |
| **Confidentiality: unable to confirm patient identity** |  |  |  | 0.038 |
| Disagree & Strongly Disagree | 55 (15.85) | 65 (23.55) | 120 (19.26) |  |
| Neutral | 107 (30.84) | 85 (30.8) | 192 (30.82) |  |
| Agree & Strongly Agree | 185 (53.31) | 126 (45.65) | 311 (49.92) |  |
| **Lack of training on telemedicine use** |  |  |  | <0.001 |
| Disagree & Strongly Disagree | 97 (27.95) | 132 (47.83) | 229 (36.76) |  |
| Neutral | 125 (36.02) | 76 (27.54) | 201 (32.26) |  |
| Agree & Strongly Agree | 125 (36.02) | 68 (24.64) | 193 (30.68) |  |
